# Family systemic therapy in patients with eating disorders

**DOI:** 10.1192/j.eurpsy.2024.1474

**Published:** 2024-08-27

**Authors:** P. Setién Preciados, C. Díaz Mayoral, E. Arroyo Sánchez

**Affiliations:** Servicio de Psiquiatría, Hospital Universitario Príncipe de Asturias, Alcalá de Henares, Spain

## Abstract

**Introduction:**

Eating disorders are a group of pathologies in which negative beliefs about food, body type and weight are associated with conducts that include food restriction, binge eating, excessive exercise, induced vomiting and the use of laxatives. They can be really severe, affecting quality of life and lead to multiple physical and psychiatric complications, even with a deadly fate.

**Objectives:**

Presentation of a patient’s case with an eating disorder and the intervention with her family, as well as, doing a review of the family interventions in these kinds of patients.

**Methods:**

Presentation of a patient’s case and review of existing literature, in regards to the use of family therapy in patients with eating disorders and its effects.

**Results:**

As in the patient’s case, there are a lot of studies that support the evidence of improvement using family therapy in patients with eating disorders. However, the difficulty to isolate the necessary variables in order to do studies about psychological treatments, complicates finding scientific evidence that supports the clinical evidence that we see in our patients day by day with these types of interventions.

**Conclusions:**

There are studies that support the efficacy of these types of family interventions. However, there needs to be a more thorough investigation with the objective of finding the more precise optimal family intervention, and specifically, determining for who and under what conditions, certain types of family interventions would be more effective.

**Disclosure of Interest:**

None Declared

